# Effects of Copper Dopants on the Magnetic Property of Lightly Cu-Doped ZnO Nanocrystals

**DOI:** 10.3390/nano10081578

**Published:** 2020-08-11

**Authors:** Zhi Wang, Wenzhen Xiao, Mengmeng Tian, Neng Qin, Haidong Shi, Xiwei Zhang, Wenke Zha, Jiahua Tao, Junlong Tian

**Affiliations:** 1 School of Physics and Electrical Engineering, Anyang Normal University, Anyang 455000, China; 01564@aynu.edu.cn (W.X.); 01566@aynu.edu.cn (M.T.); 01551@aynu.edu.cn (N.Q.); 02008@aynu.edu.cn (H.S.); zhangxw@aynu.edu.cn (X.Z.); science@hnu.edu.cn (W.Z.); tjl@aynu.edu.cn (J.T.); 2 Key Laboratory of Polar Materials and Devices (MOE), East China Normal University, Shanghai 200241, China; jhtao@phy.ecnu.edu.cn

**Keywords:** diluted magnetic semiconductors, Cu doped ZnO, photoluminescence, ferromagnetism

## Abstract

To explore the origin of magnetism, the effect of light Cu-doping on ferromagnetic and photoluminescence properties of ZnO nanocrystals was investigated. These Cu-doped ZnO nanocrystals were prepared using a facile solution method. The Cu^2+^ and Cu^+^ ions were incorporated into Zn sites, as revealed by X-ray diffraction (XRD) and X-ray photoelectron spectroscopy (XPS). At the Cu concentration of 0.25 at.%, the saturated magnetization reached the maximum and then decreased with increasing Cu concentration. With increasing Cu concentration, the photoluminescence (PL) spectroscopy indicated the distribution of V_O_^+^ and V_O_^++^ vacancies nearly unchanged. These results indicate that Cu ions can enhance the long-range ferromagnetic ordering at an ultralow concentration, but antiferromagnetic “Cu^+^-Vo-Cu^2+^” couples may also be generated, even at a very low Cu-doping concentration.

## 1. Introduction

The combination of logic semiconductors with the information storage capabilities of magnetic elements can be realized possibly in a single material through spintronic devices. Fortunately, room temperature ferromagnetism (RTFM) can often be observed in ZnO semiconductor material doped by transition metals (TM) [1,2]. Therefore, zinc oxide (ZnO) doped by 3*d* TM (e.g., Co [3], Ni [4], Mn [5,6], Cu [7], and Cr [8]) has been investigated extensively as a promising diluted magnetic semiconductor (DMS) for prospective applications in spintronic devices. However, the debates on the origin of ferromagnetic behavior and coupling mechanism are still open, which have been attributed to metal clusters, vacancy clusters [9,10], secondary oxide phases [11,12], and magnetic contamination [13].

Cu-doped ZnO system has been given much attention due to two reasons given below. Firstly, the radius match-up of Cu and Zn ions can lead to a low formation energy. Secondly, neither metallic Cu nor copper oxides (CuO and Cu_2_O) are ferromagnetic [14]. For these reasons, there is an opportunity to clarify the origin and the coupling mechanism of ferromagnetic behavior of diluted magnetic semiconductors, which are still under debate [2,15]. Upon this, much effort has been devoted to investigating Cu-doped ZnO systems. More recently, more meaningful phenomena were discovered upon Cu-doped ZnO. For example, Liu et al. reported that RT polarization-applied electric field (P-E) loops can be observed directly, demonstrating the existence of multiferroic properties of the ZnO:Cu films [16]. Muhammad Younas found that the resistance can be switched from high resistance (HRS) to low resistance (LRS) through reversible tuning of the ferromagnetism of Cu-doped ZnO thin films [17]. These results have made Cu-doped ZnO even more appealing to researchers.

However, there is no consensus on the origin of RTFM. Sharma et al. reported that RTFM of Cu-doped ZnO nanorods decreased with increasing Cu concentration (1–5%) [18], while Yıldırım et al. found that RTFM increased [14]. The electronic structure of Cu-doped ZnO has been calculated, indicating that the empty Cu 3*d* states are located in the gap region without the hybridization with the Zn 4*s* conduction band due to the strong Cu-O covalency in CuO_4_ tetrahedron [19]. This would make the Cu solubility in ZnO very low (~1% at most). It means that secondary oxides can be formed when the Cu doping level exceeds 1%, and this has been verified experimentally [20]. Further, some results found that secondary oxides and its related structures of Cu dopants can also contribute to the RTFM [12,21], which is a discrepancy from that reported before [22]. Hou et al. found that porous Cu_2_O thin films manifest unexpectedly large RTFM, which was ascribed to coupling between oxygen vacancies and local magnetic moments [21]. Gao et al. reported that the RTFM of the CuO-ZnO system can be tuned by the interface counts for CuO and ZnO heterostructures [12]. Thus, it is necessary to lower the concentration of Cu dopants to prevent the generation of Cu-related secondary phases, minimizing the effect of extrinsic sources on the RTFM.

Herein, we prepared a series of lightly Cu-doped ZnO nanocrystals (~1% at most) using a facile one-pot solution method. The ferromagnetic property was characterized by measuring the field-dependent magnetization (M–H) curves of these nanocrystals. The role of Cu dopants in the magnetic property of lightly Cu-doped ZnO nanocrystals was investigated with the help of X-ray diffraction (XRD), X-ray photoelectron spectroscopy (XPS), and photoluminescence (PL) spectroscopy. Our results suggest that Cu ions at an ultralow concentration can enhance the long-range ferromagnetic ordering but may generate the antiferromagnetic “Cu^+^-Vo-Cu^2+^” couples even at a very low Cu doping concentration.

## 2. Experimental Section

### 2.1. Chemicals

Zinc acetate dihydrate (Zn(OOCCH_3_)_2_·2H_2_O), hexamethylenetetramine (HMTA), and copper acetate monohydrate (Cu(OOCCH_3_)_2_·H_2_O) were purchased from Sigma-Aldrich Co. LLC. (Shanghai, China). All the chemicals are of spectrography grade or better and used without further purification.

### 2.2. Preparation of Lightly Cu-Doped ZnO

A serial of lightly Cu-doped ZnO with various Cu concentrations were prepared by a facile solution method. In a typical synthesis procedure, stoichiometric Zn(OOCCH_3_)_2_·2H_2_O and Cu(OOCCH_3_)_2_·H_2_O (total up to 0.03 mol) were dissolved in 300 mL deionized water as the precursor together with 0.03 mol HMTA. The mixture was ultrasonicated and stirred vigorously for 2 h in a beaker. Before growth, a glass substrate was placed in the beaker. The glass substrate was cleaned by a piranha solution for 10 min and followed by deionized water under ultrasonication [23]. Then, the mixture were sealed and kept at 95 °C for 24 h. After growth, the substrate was rinsed in deionized water thoroughly and dried in air at room temperature. The stoichiometric ratios of Cu(OOCCH_3_)_2_·H_2_O to Zn(OOCCH_3_)_2_·2H_2_O were set to 0, 0.1%, 0.25%, 0.5%, and 1%, respectively.

### 2.3. Characterizations

X-ray diffractions of the samples were carried out using a Ni-filtered Cu K_α_ (λ = 1.5418 Å) radiation source (D/Max-2550 V, Rigaku Co., Tokyo, Japan). The XRD scans were collected from 30° to 80° (2Θ), with a step of 0.02° and a data collection time of 0.5 s. The JCPDS PDF database [24] was utilized for phase identification. All the products underwent scanning electron microscopy (SEM, JSM7500SF, JEOL, Japan) to compare the morphology of these samples. X-ray photoelectron spectroscopy (XPS) was conducted in an Axis Ultra DLD spectrometer using a monochromatized Al K_α_ X-ray source (1486.6 eV) in order to monitor the binding state of the ions. Magnetic measurements were performed on a Superconducting Quantum Interference Device (SQUID, MPMS-2, Quantum Design Inc., San Diego, CA, USA). The samples were filled in a small container made of polyvinyl chloride, whose diamagnetic moment was subtracted from the measured magnetization values. The photoluminescence spectra were recorded at room temperature with a Jobin-Yvon LabRAM HR 800 UV micro-PL spectrometer using He-Cd (325 nm) laser as the excitation source.

## 3. Results and Discussions

**Figure 1:**
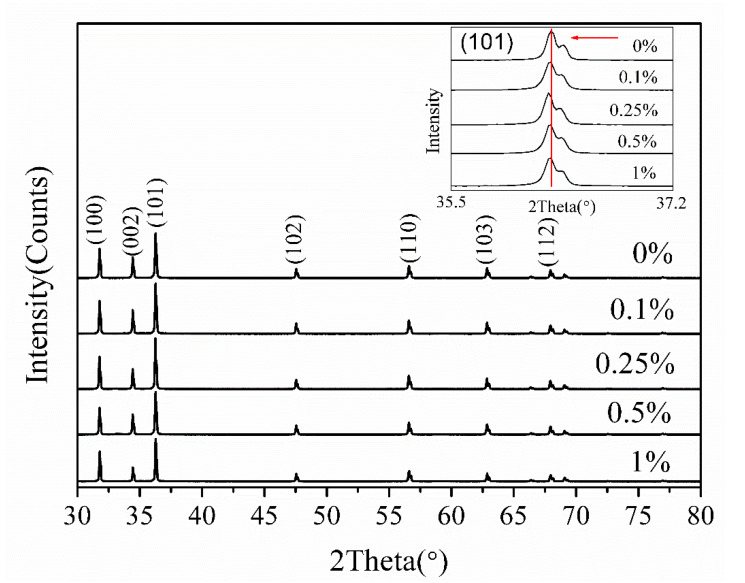
XRD spectra of undoped ZnO and lightly Cu-doped ZnO with concentrations of 0.1%, 0.25%, 0.5%, and 1%. The inset is the magnified patterns of (101) crystal direction.

Figure 1 presents the X-ray diffraction (XRD) *θ*–2*θ* scan of as-grown undoped ZnO and lightly Cu-doped ZnO nanocrystals. All the diffraction peaks can be readily indexed as a hexagonal wurtzite ZnO structure (JCPDS card No. 36-1451) with space group P6_3_mc and lattice constants of *a* = 3.250 Å and *c* = 5.207 Å. No other peaks corresponding to copper and its related secondary phases were detected within the detection limit of XRD. This reveals that the substitution of Cu dopants does not change the wurtzite structure. The inset in Figure 1 shows the magnified (101) peaks of XRD patterns. A tiny blue shift of the (101) peaks of these three lightly Cu-doped ZnO nanocrystals can be discerned by comparing with the undoped ZnO sample, indicating that the lattice constants were enlarged due to the substitution of Cu dopants.

**Figure 2:**
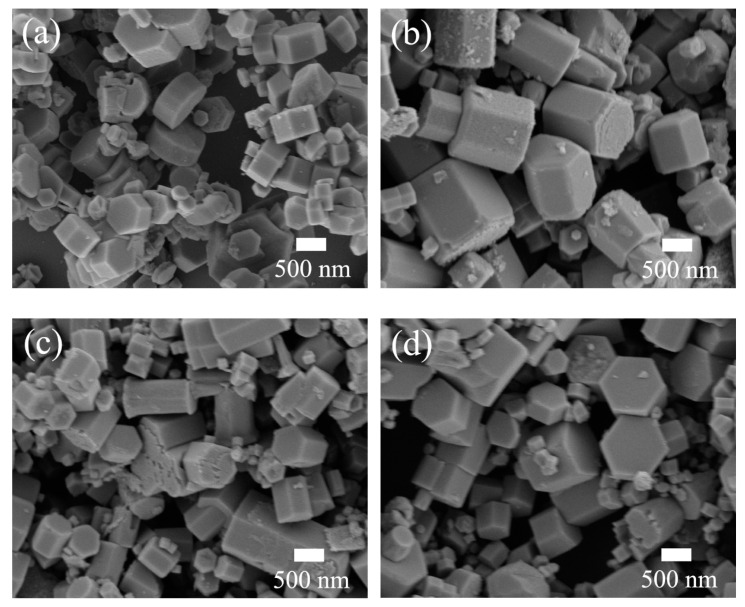
SEM images of undoped ZnO (**a**) and lightly Cu-doped ZnO nanocrystals with different Cu concentrations: (**b**) 0.25%, (**c**) 0.5%, and (**d**) 1%.

To study effects of Cu concentration on the morphology of lightly Cu-doped ZnO samples, SEM measurement was performed. The results are represented in Figure 2. In the absence of Cu dopants, the morphologies of the obtained undoped ZnO nanocrystals were nanoprisms which were not uniform. The diameter and thickness of the nanoprisms were in a range of 310–800 nm and 200–400 nm, respectively, as shown in Figure 2a. After the addition of Cu dopants with a molar ratio of 0.25%, the nanoprisms were relatively more uniform and the thickness of the nanoprisms increased to a range of 400–1000 nm with the diameter nearly unchanged (Figure 2b). With a further increase of the molar ratio of Cu dopants to 0.5%, the diameters of the nanoprisms tapered to a range of 250–500 nm (Figure 2c). When the molar ratio of Cu dopants reached 1%, the diameters of the nanoprisms increased to the range of 400–1000 nm again as shown in Figure 2d. The SEM results illustrate that the aspect ratio of ZnO nanocrystals can be modulated by Cu dopants, because of which the rate of nucleation and subsequent growth can be influenced. Similar results were also reported in Cu-doped ZnO nanocrystals prepared via the vapor transport method [25] and hydrothermal method [18].

**Figure 3:**
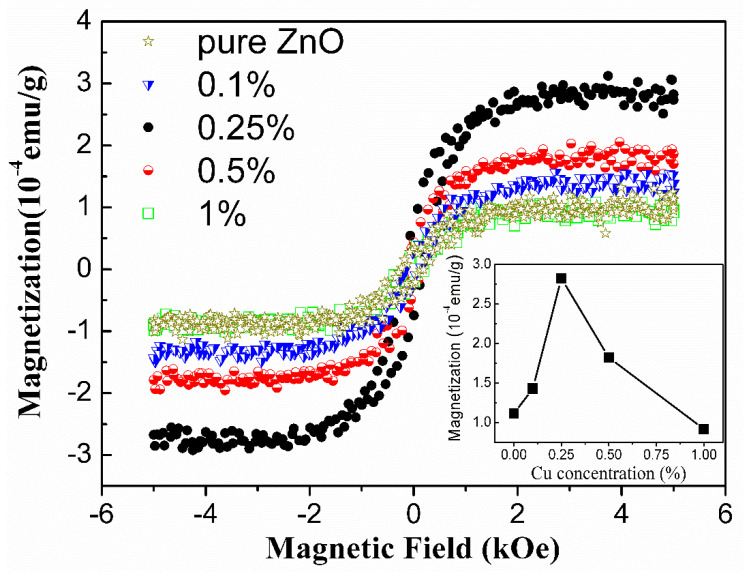
The room temperature field-dependent magnetization (M–H) curves of undoped and lightly Cu-doped ZnO nanocrystals with different Cu concentrations.

The magnetic properties of these undoped ZnO and lightly Cu-doped ZnO nanocrystals were characterized by a Superconducting Quantum Interference Device (SQUID) magnetometer. The field-dependent magnetization (M–H) curves measured at room temperature in a field range of 0 ± 5 kOe are shown in Figure 3. It can be seen that the magnetization of all the samples becomes saturated at ca. 2 kOe. The variation tendency of the saturated magnetization was depicted in the lower right corner of Figure 3. By increasing the Cu concentration, the saturated magnetization of Cu-doped ZnO nanocrystals increased quickly from the value of 1.11 × 10^−4^ to 2.83 × 10^−4^ emu/g (0.25 at.%) and then decreased linearly to the value of 0.92 × 10^−4^ emu/g (1 at.%). The saturated magnetizations are comparable with the results reported in the literatures [26,27]. The temperature-dependent magnetization (M–T) were also measured and are shown in Figure S1. In the DMS field, Ram Seshadri pointed out that the magnetic signals of this size (~10^−6^ emu, assuming sample mass 10 mg) were often misattributed, and time and again shown to be due to impurities from sample holders, oxide phases, etc. [28]. In our case, extrinsic effects such as oxide phases can be ruled out. On one hand, the Cu-doping concentrations in Figure 3 are not more than the threshold (1%) predicted by Part et al. [19]. On the other hand, if the observed ferromagnetic phenomenon were caused by oxide phases, the trend should be linear with increasing the doping concentration since more oxide phases could be formed by increasing the doping concentration. Even for the 1.5% Cu-doped sample shown in Figure S2, the ferromagnetic signal also shows a consistent trend observed in our case. To investigate the effect of sample holders, the M–H curves of the blank sample holder and those observed after a subtraction of the diamagnetic background were measured and are shown in Figure S3. It can be seen that the blank sample holder shows nearly a diamagnetic behavior, and the small ferromagnetic signals were ~10^−7^ emu. Compared to our smallest ferromagnetic moment (10^−4^ emu/g, sample mass ~50 mg), shown in Figure 3, the effect of the sample holder on these samples with Cu concentration not more than 1% can be ignored. These data demonstrate that the RTFM of lightly Cu-doped ZnO nanocrystals can be indeed modulated by the Cu dopants.

**Figure 4:**
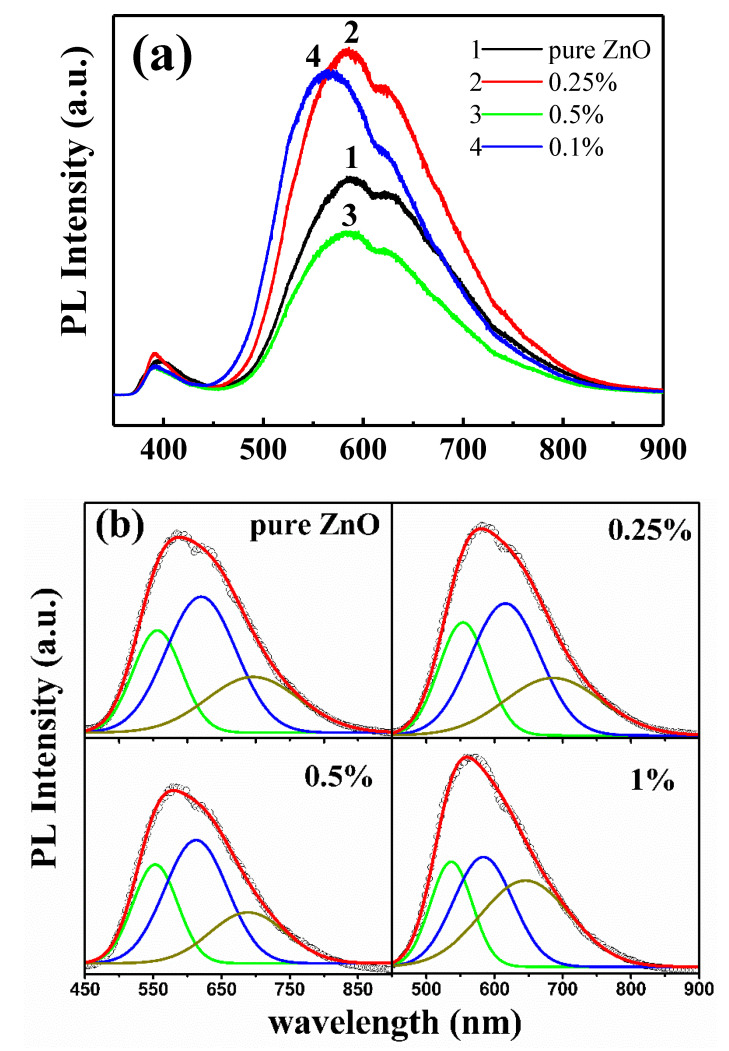
Room temperature (RT) photoluminescence spectra of undoped ZnO and lightly Cu-doped ZnO nanocrystals with different concentrations (**a**) and Gaussian fitted Curves (**b**) for these experimental spectra.

Although the real origin of RTFM in Cu-doped ZnO system is intriguing and not clearly understood, an emerging consensus is coming that ferromagnetism of Cu-doped ZnO system can be surely induced or mediated by defects in the crystals [29,30]. Therefore, to investigate the defects’ distribution in the lightly Cu-doped ZnO nanocrystals, photoluminescence measurement was carried out. As shown in Figure 4a, all the samples show two distinct emission peaks: a sharp one in the ultraviolet (UV) region and another broad one in the visible region. The former, centered at 388–395 nm, is attributed to free excitonic near-band-edge emission, while the latter is mainly referred to recombination of electrons deeply trapped in oxygen/Zn vacancies and interstitials with photogenerated holes [31]. Much work has been devoted to investigating the origin of the PL in the visible range [32,33,34,35]. Generally, the green emission around 527 nm is mainly attributed to the recombination of electrons trapped in single ionized oxygen vacancies (V_O_^+^) with photogenerated holes [32,33]. The green emission around 575 nm is ascribed to the doubly ionized oxygen vacancy (V_O_^++^) [32,34]. The red emission around 657 nm originates from the intrinsic defects of oxygen interstitials (O_i_) [36]. To carefully study the above three kinds of defects in ZnO:Cu nanocrystals, Gaussian fitting was performed on the visible band in the range of 450–900 nm (Figure 4b). With the Cu concentration increasing, the concentration of V_O_^+^ nearly remained unchanged since the Cu concentrations were too low to significantly influence the defect contribution (the defect concentration was calculated from the integral area). Many works have proven that RTFM of pure ZnO and TM-doped ZnO can be modulated through changing the concentration of oxygen vacancies, especially singly ionized oxygen vacancies by post-annealing ZnO samples in different atmospheres [30,31,37]. However, in this work, RTFM varies remarkably with increasing Cu concentration in the low doping level (~1% at most) while defects’ distribution is nearly the same.

**Figure 5:**
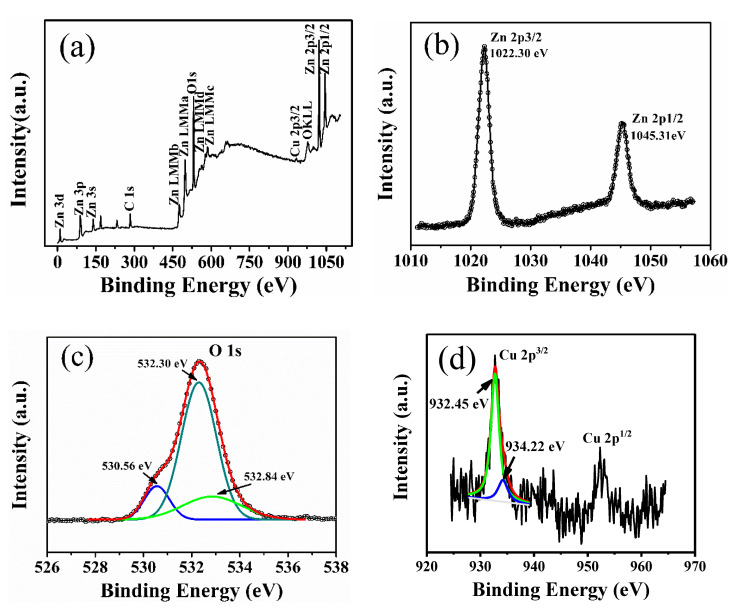
XPS spectra of lightly Cu-doped ZnO (0.25%) nanocrystals: (**a**) Wide-scan spectrum; (**b**–**d**) the corresponding narrow-scan spectra of Zn 2*p*, O 1*s*, and Cu 2*p*, respectively. Peak positions are referenced to the adventitious C 1*s* peak taken to be at 284.6 eV.

To further clarify the origin of RTFM, XPS measurement was performed to investigate the bonding characteristics and oxidation states of Cu-doped ZnO nanocrystals with a molar ratio of 0.25%. The results are represented in Figure 5. The full-range survey scan XPS spectrum in Figure 5a demonstrates that the only elements of the wurtzite-type lightly Cu-doped ZnO crystals are Zn, O, and Cu. Figure 5b shows the high-resolution Zn 2*p* core-level XPS spectrum. The double symmetrical peaks located at 1022.30 and 1045.31 eV with a difference of 23.01 eV can be ascribed to Zn 2*p*_3/2_ and Zn 2*p*_1/2_, respectively [38,39], revealing that the oxidation of the Zn atoms is the Zn^2+^ chemical state. The broad and asymmetric O 1*s* spectrum can be well fitted by three Gaussian curves as depicted in Figure 5c. The lower binding energy peak centered at 530.56 eV is attributed to O^2−^ ions in wurtzite structure of hexagonal ZnO [40]. The strongest peak with a higher binding energy centered at 532.30 eV is assigned to O^2−^ ions in the oxygen-deficient regions within the matrix of ZnO [39], indicating that plenty of oxygen deficiencies exist on the surface of lightly Cu-doped ZnO nanocrystals, which is consistent with the result revealed by the PL spectra shown in Figure 4. The highest binding energy peak centered at 532.84 eV can be attributed to the adsorbed oxygen molecular on the surface of the nanocrystals [41]. Figure 5d represents the high-resolution XPS spectrum of Cu 2*p* located at 920–970 eV. As the Cu-doped level is very low, only the peak of Cu 2*p*_3/2_ is well-shaped. The asymmetrical peak of Cu 2*p*_3/2_ was also fitted by two Gaussian curves centered at 932.45 eV for Cu^1+^ (*d*^10^) and 934.22 eV for Cu^2+^ (*d*^9^), respectively [16,42]. This obviously shows that the Cu ions are of two valent states, and the nonmagnetic Cu^1+^ (*d*^10^) ions account for 86% of all the Cu ions. The corresponding narrow-scan spectra of Cu 2*p* in 0.1%, 0.5%, and 1% Cu doped samples were also measured and are shown in Figure S4. For 0.5% and 1% Cu-doped samples, the nonmagnetic Cu^1+^ (*d*^10^) ions account for 83% and 80%, respectively. This result is in coincidence with the XRD results (Figure 1). The inset of Figure 1 indicates that the lattice constants of ZnO were enlarged by the doped Cu ions. It is known that the radius of Cu^+^ (0.77 Å) ion is bigger than that of Zn^2+^ (0.74 Å) and Cu^2+^ (0.73 Å) ion [43]. Therefore, it is reasonable to assume that the lattice constants should be enlarged when the Zn^2+^ ions are substituted by Cu^+^ ions. As is known, all electrons of Cu^+^ ion are paired in the 3*d*^10^ configuration, and hence, Cu^+^ ions cannot produce a magnetic moment [44]. Therefore, only Cu^2+^ ions accounting for a small percentage are responsible for the observed RTFM.

**Figure 6:**
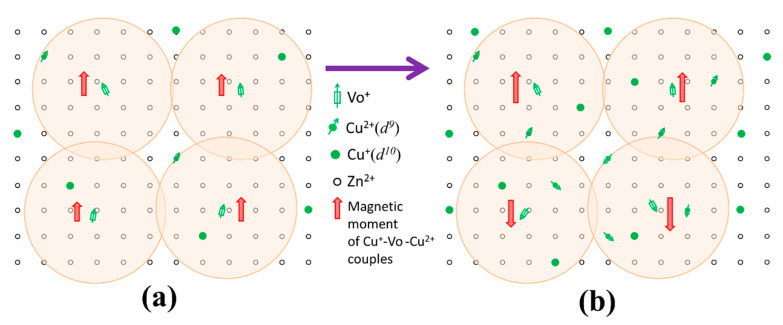
The schematic illustration of the magnetic moment evolution with increasing Cu concentration of lightly Cu-doped ZnO: (**a**) ferromagnetism of Cu^+^-V_O_-Cu^2+^ couples through an indirect double-exchange model; (**b**) antiferromagnetic Cu^+^-V_O_-Cu^2+^ couples dominated by Cu ions. The orange circle represents the oxygen vacancy orbital.

Grounded on the above PL observations and the structural properties presented in Figure 1 and Figure 5, we go back to the magnetic properties of the as-prepared samples in Figure 3, which show that the saturated magnetization reached the maximum at the Cu concentration of 0.25 at.% and then decreased with Cu concentration increasing to 1%. The RTFM in undoped ZnO is generally attributed to oxygen vacancies (V_O_) which can initiate defect-related hybridization at the Fermi level and establish a long-range ferromagnetic ordering [30,45]. When Cu ions were incorporated into Zn sites, Herng et al. pointed out that both oxygen vacancies (V_O_) and Cu dopants contributed to RTFM through an indirect double-exchange model, e.g., Cu^+^-V_O_-Cu^2+^ [46]. In this model, Cu dopants are mediated by the Vo orbital which has a large size with a radius of about 2.25 times the lattice constant. The Cu ions with different states coupled ferromagnetically by virtual hopping of the extra electron from one ion to another via V_O_ orbital. Noteworthy, the Cu^2+^ ions outside the range of Vo vacancies would remain magnetically disordered due to the lack of the indirect double-exchange coupling channel, and only Cu^2+^ ions residing in the range of Vo vacancies can couple ferromagnetically in this model. This means that only a small fraction of the Cu^2+^ can contribute to the observed ferromagnetic signal. In our case, the Cu concentration was very low and the effect of impurity phases can be ruled out. Cu ions were incorporated into Zn sites indicated by XRD results (Figure 1). Further, our XPS measurement reveals that Cu ions were of two valent states and the proportion of the nonmagnetic Cu^1+^ (*d*^10^) ions was not less than 80% of all the Cu ions in all the Cu doped samples. This is also consistent with the results (83%) reported in [46] that the Cu dopants in the proximity of Vo orbital would behave more like *d*^10^ (Cu^1+^-like) when receiving the doped electrons. In this Cu-doped ZnO system, both oxygen vacancies and Cu dopants can contribute to RTFM through an indirect double-exchange model. However, our PL results demonstrate that the oxygen deficiency distributions remained nearly unchanged with increasing Cu concentration under low doping level. Therefore, it can be concluded that Cu dopants and their coupling with oxygen deficiencies are responsible for the observed phenomenon. Based on the above analysis, a possible mechanism for the magnetic moment evolution with increasing Cu concentration is suggested, as illustrated in Figure 6. At the initial state, the doping of Cu^2+^ ions can couple with Vo^+^ ferromagnetically through indirect double-exchange model and enhance the long-range ferromagnetic ordering since magnetic Cu^2+^ ions were introduced into ZnO wurtzite structure (Figure 6a). With increasing Cu dopant concentration, the distance between two Cu ions becomes shorter and shorter. Feng et al. found that antiferromagnetic configuration was favored over the ferromagnetic one when the distance between Cu ions was in a certain range based on the B3LYP hybrid density functional [47]. Therefore, it is reasonable to propose that with further increasing Cu concentration beyond 0.25% continuously at low doping level (~1% at most), the distance between Cu ions became shorter and the antiferromagnetic couples were dominated by the Cu ions generated, resulting in the declination of the magnetization (Figure 6b). This antiferromagnetic phenomenon was also reported in other TM-doped ZnO systems, such as Co [48], Ni [49], and Mn [50,51]. Further work needs to be done to study the coupling behavior between the magnetic dopants and defects on the ferromagnetism of Cu-doped ZnO system at low doping level.

## 4. Conclusions

We have found that room temperature ferromagnetism observed in lightly Cu-doped ZnO nanocrystals could be modulated by changing the concentration of Cu dopants. Combining the XRD and XPS analysis, Cu^2+^ and Cu^+^ ions were successfully incorporated into Zn sites of ZnO wurtzite structure. Oxygen deficiencies were abundant in the lightly Cu-doped ZnO nanocrystals, and the distribution of these defects was nearly unchanged, as revealed by XPS and PL measurements. The Cu dopants are responsible for the variation of room temperature ferromagnetism. The results suggest that Cu ions at an ultralow concentration can enhance long-range ferromagnetic ordering but may generate antiferromagnetic “Cu^+^-Vo-Cu^2+^” couples even at a very low Cu-doping concentration.

## Supplementary Materials

The following are available online at https://www.mdpi.com/2079-4991/10/8/1578/s1, Figure S1: The M–T curves of the 0.25% Cu doped ZnO sample, Figure S2: The M–H curves of the 1.5% Cu doped ZnO sample and those after a subtraction of the diamagnetic background, Figure S3: The M–H curves of the blank sample holder and those after a subtraction of the diamagnetic background, Figure S4: The corresponding narrow-scan spectra of Cu 2*p* for the 0.1%, 0.25%, 0.5% and 1% Cu doped samples.
