# Supplementary material for: Effects of Copper Dopants on the Magnetic Property of Lightly Cu-Doped ZnO Nanocrystals
Source: Nanomaterials (Basel). 2020 Aug 11;10(8):1578. doi: 10.3390/nano10081578 (PMC7466550; doi:10.3390/nano10081578)
Supplement: Supplementary file 1 [file nanomaterials-10-01578-s001.pdf]

Article

Effects of Copper Dopants on the Magnetic Property of Lightly Cu-Doped ZnO Nanocrystals

Zhi Wang, Wenzhen Xiao, Mengmeng Tian, Neng Qin, Haidong Shi, Xiwei Zhang, Wenke Zha, Jiahua Tao and Junlong Tian

Supplementary Materials

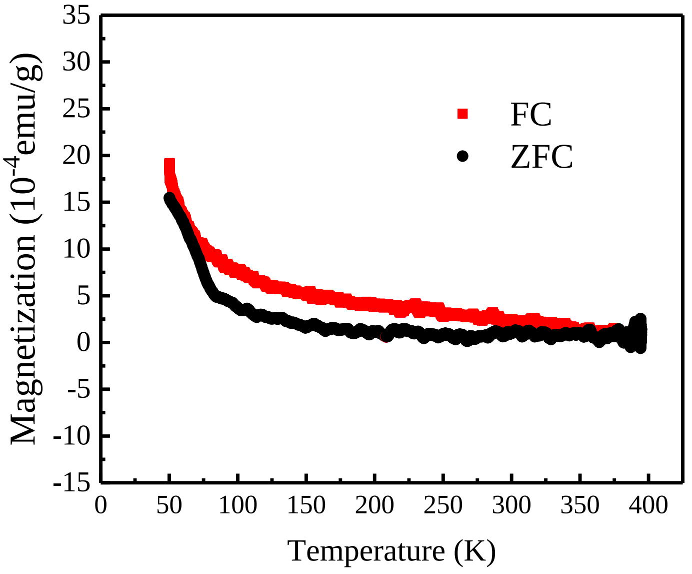

Figure S1. The M–T curves of the 0.25% Cu-doped ZnO sample.

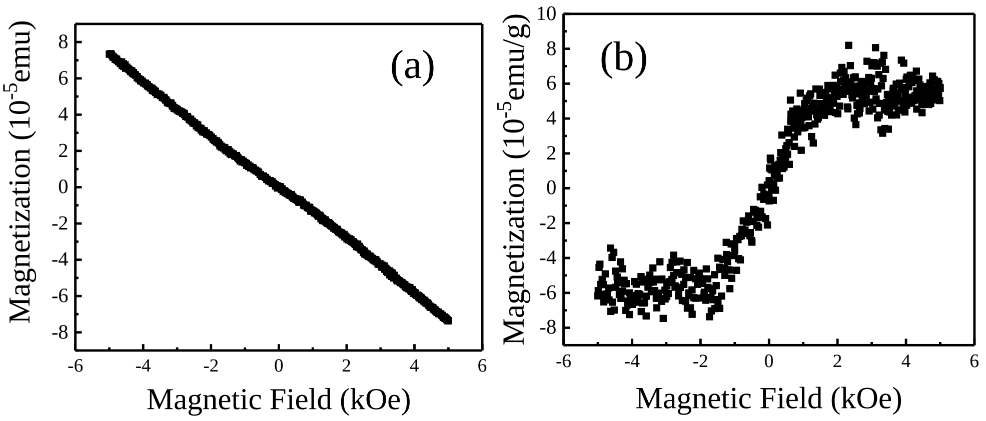

Figure S2. The M–H curves of the 1.5% Cu-doped ZnO sample and those after a subtraction of the diamagnetic background.

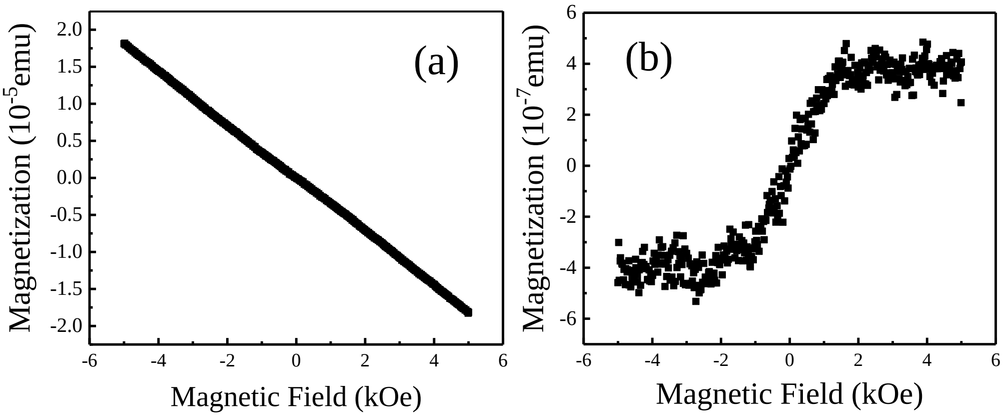

Figure S3. The M–H curves of the blank sample holder and those after a subtraction of the diamagnetic background.

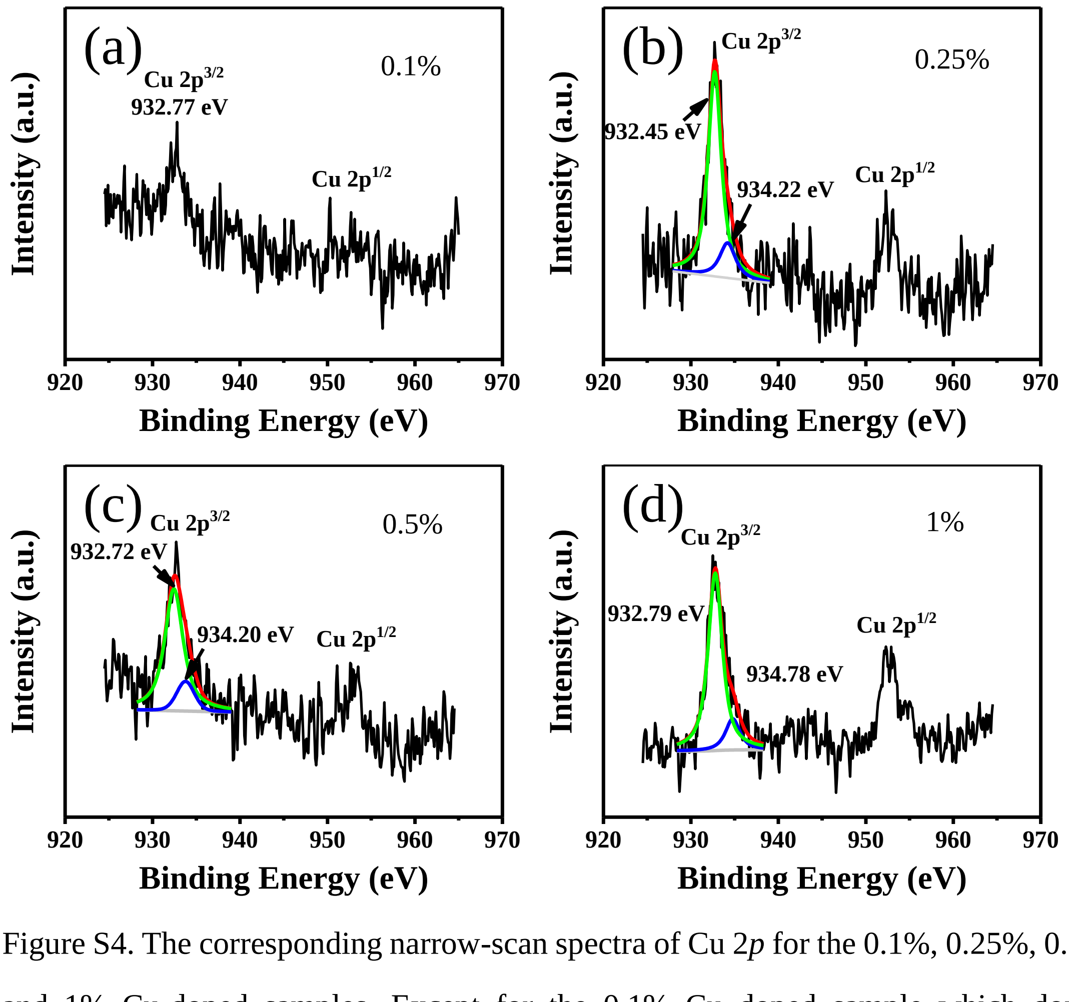

Figure S4. The corresponding narrow-scan spectra of Cu 2*p* for the 0.1%, 0.25%, 0.5% and 1% Cu-doped samples. Except for the 0.1% Cu doped sample which doped concentration is too low, the Cu XPS peaks are well-shaped and can be fitted by Cu^{2+} and Cu^{1+} phases. The nonmagnetic Cu^{1+} (d^{10}) ions accounts for 86%, 83% and 80% for the 0.25%, 0.5% and 1% Cu-doped samples, respectively.
